# “Replace them by Salads and Vegetables”: Dietary Innovation, Youthfulness, and Authority, 1900–1939

**DOI:** 10.1080/20549547.2018.1460538

**Published:** 2018-04-23

**Authors:** James F. Stark

**Affiliations:** a School of Philosophy, Religion and History of Science, University of Leeds, Leeds, UK

**Keywords:** Vitamins, diet, fasting, aging, youth, rejuvenation

## Abstract

The events of the First World War fueled public fascination with rejuvenation at the same time as medical scientists began to explore the physiological potential of so-called “vitamine.” The seemingly bottomless capacity of vitamins to maintain bodily function and appearance offered a possible mechanism for achieving bodily renewal, alongside established dietary practices such as abstention from alcohol and meat. Drawing on mainstream medical publications, popular dietary texts and advertising materials, this paper outlines how vitamins and other dietary practices played an important but hitherto unrecognized role in reconfiguring ideas about anti-aging and rejuvenation. I argue that new ways of understanding food and its relationship with the body were at the heart of attempts by various groups to claim expertise about and authority over diet and its effects, not just on the human body in general, but on the aging process in particular.

## Introduction: “A Lack of Vitamines in the Dietary”

In 1919 the UK Medical Research Council (MRC) published a major report on what were then known as “accessory food factors” or “vitamines.”^1^ The group charged by the MRC with investigating the role of these vitamines was led by the eminent English biochemist Frederick Gowland Hopkins. Hopkins held a long-standing interest in cellular metabolic pathways, having speculated in public as early as 1906 that as-yet-undiscovered “accessory food factors” had an important role in the high-profile nutritional deficiency diseases of rickets and scurvy.^2^ In 1912 he expanded the supposed role of these factors into developmental biology when he was among the first to theorize that the major constituents of food – proteins, carbohydrates, fats, minerals and water – were not by themselves sufficient to enable normal animal growth. Given this, Hopkins argued that there must be some additional component or components which were essential in ensuring correct metabolic function and assimilation of food.^3^

His influence on the field in Britain can be seen in the fact that the term vitamines – favored by another pioneer of vitamins, Casimir Funk, and promoted in Funk’s 1912 book *The Vitamines* – was relegated in the report at the expense of Hopkins’ preferred description: “accessory food factors.”^4^ Subsequent British authors used the terms interchangeably throughout the interwar period.^5^ Indeed, this is all the more surprising given that Hopkins had hardly published on the subject after 1912; he nevertheless remained an important figurehead for vitamin science in Britain.^6^ The report, led by Hopkins, but very much a collaborative endeavor, made clear that there was “at present no knowledge concerning … [the] actual chemical nature” of vitamines, although they appeared to act on the body out of all proportion with the amount actually consumed.^7^ The overwhelming view was that these vitamines were powerful agents in the body, essential for the maintenance of normal physiological function.

In the longer term, Hopkins was frustrated at his lack of progress in unlocking the structure and function of vitamins.^8^ However, his role in promoting the physiological significance of vitamins should not be underestimated. At the same time as he and his fellow authors were poring over the factors underlying diseases of dietary deficiency such as rickets, pellagra, beri-beri and scurvy, public audiences in Britain were being offered tantalizing glimpses into a future where medical science might be able to slow, stop or even reverse the aging process. The 1920s, when vitamins were trumpeted from various quarters as a form of dietary salvation, has been characterized by Chandak Sengoopta as “the decade of rejuvenation” when expensive and exclusive new hormone-based procedures designed to restore lost vitality captured the spirit of the age and the attention of public and professional audiences alike.^9^

As well as the expensive and (for most people at least) inaccessible surgical interventions practiced by prominent hormone rejuvenators including Eugen Steinach and Serge Voronoff, however, a raft of everyday products and programs which claimed to have rejuvenating potential flooded the marketplace. Common to many of these elixirs, tonics, skin creams and devices were new claims about the potential anti-aging properties of various foodstuffs. By the advent of the Second World War, advertisements for new vitamin-containing dietary supplements brought together the language of vital force and rejuvenation, claims about the natural origins of active ingredients and appeals to scientific purity and authority. Specific vitamins were now reputed to have specific pathways of action, interacting with the endocrine system and the natural physiology of the body to produce anti-aging effects. Other methods, including more general modifications in diet and periodic, so-called “rational” fasting were championed by writers who sought to capitalize on the emerging public fascination with rejuvenation.^10^

Although early scientific research into vitamins and their function has received considerable historical attention this has largely concentrated on their role in healthy diets, links with the commercialization of foodstuffs and the emergence of new sciences of nutrition.^11^ Much of this has focused on the United States and the so-called “food reforms” of the first two decades of the twentieth century following a more general program of liberal reform in the period from 1906–1914.^12^ There was overwhelming scientific consensus that vitamin substances had a profound effect on human physiology, mediated through metabolism, yet we still know comparatively little about how these powerful agents of bodily control were thought to interface with that object of interwar fascination: aging. Appreciating now that the theme of rejuvenation was one of tremendous public interest in this period, across national boundaries, allows us to view vitamins and dietary practices more generally in a new light.^13^

Despite the hugely significant interplay between the discovery of vitamins, new kinds of dietary innovation and the aging process, and an established body of literature examining aging and old age, historians have yet to interrogate this relationship.^14^ The core motivating question underpinning this paper arises at the intersection of these. In an era of widespread anxiety about the health, fertility and fitness of both individuals and nations, how were innovations in dietary science and practice presented by medical scientists and manufacturers as ways of achieving rejuvenation? This paper outlines a relationship between the social effects of the First World War on perceptions and practices of age, youthfulness and dietary innovation. In the years immediately following this global conflict, the vitamin content of various foods was widely heralded by publics and professionals as a potentially defining feature of a new era of physical and mental fitness in Britain, as the country sought to respond to David Lloyd George’s assertion that “you cannot maintain an A 1 Empire with a C 3 Nation” and come to terms with an aging population.^15^

Drawing on mainstream medical literature, popular dietary texts, and advertising materials, I argue that strategic consumption of vitamin-rich foods and, later, vitamin extract products, as well as long-established dietary practices, such as fasting, became an important part of strategies designed to maintain youthfulness, health and vitality.^16^ In a period when other possible methods of rejuvenation, such as hormone treatments, electrotherapy and exercise regimes, fueled a public imperative to maintain vitality, increasingly scientized dietary practices were promoted to consumers as an easy alternative.^17^

The story is centered primarily, although not exclusively, on the Anglo-American context, though products and those who promoted them were not nationally bound.^18^ Anxious agers and eugenicists alike were captivated by the possibility of prolonging youth, while the seemingly bottomless potential of vitamins to maintain youthful bodily function and appearance offered a possible mechanism for achieving these goals, alongside established dietary practices such as abstention from alcohol and meat.

## Building the Young Body: Pre- and Post-vitamins

In order to appreciate how the emergence of vitamin science changed dietary advice which promised rejuvenating results, we must first understand how such advice was configured in the pre-vitamin era. In his book *Old Age: Its Causes and Prevention* (1912), the American anti-aging advocate, author, and businessman Sanford Bennett proclaimed that a specific regime of exercise and diet had “thrown off the conditions of age” and enabled him to “become, physically, a young man again.”^19^ Bennett’s account was subtitled *The Story of an Old Body and Face Made Young*, highlighting his claim that modification of the diet and a strict regime of exercises – “muscular contractions” – could result in a literal restoration of youth. Drawing on his earlier 1907 publication, *Exercising in Bed*, he railed against “dangerous allopathic methods,” instead raising a “plea for Nature’s methods of cure.”^20^ The system of eating proposed by Bennett drew on a diverse range of sources, from the writings of Luigi Cornaro (c.1467–1566), a Venetian nobleman who wrote treatises on the long life, to the more recent works of Arnold Lorand (1865–1943), physician at the Carslbad Spa and advocate of everyday rejuvenation. Lorand himself was cautious about the use of extensive fasting and the omission of food groups from the diet. As he argued, “true vegetarians frequently present a pale, unhealthy and prematurely aged appearance.”^21^

Bennett’s dietary method was essentially twofold: he advised periodic and total fasting, followed by a vegetarian diet characterized by “well-cooked vegetables.”^22^ Standard components such as fat were useful for achieving an “increase of fatty tissues,” yet even Bennett did not believe “that life can be prolonged to extraordinary ages by any particular article of food.”^23^ His own personal experience was critical in establishing his authority in matters of diet; *Old Age* abounded with explicit references to “twenty-five years of experiment upon my own body” and the rejection of “all medicines.”^24^

Sanford Bennett was one of the most high-profile advocates of rejuvenating methods in the twentieth century prior to the First World War, and his exercises, promising rejuvenation at an old age, were hugely popular, particularly in the United States, as he embodied the powerful cultural cache of the physical culture movement, then at its height.^25^ Paradoxically, he reveled in his “young boy appetite,” yet at the same time castigated excessive consumption in noting that “we all eat too much.”^26^ Others, including the prominent physical culture advocate, Eugen Sandow, who had earned his fame in his native Germany as a bodybuilder, joined the call for dietary intervention in response to generalized anxieties about degeneration and obesity which accelerated from the 1880s.^27^ He traveled extensively and, although his work was particularly influential in Britain, built up an international audience across Europe and North America by staging spectacular strongman shows and through his popular periodical, *Sandow’s Magazine of Physical Culture* (1898–1907), which enjoyed notable yet brief success as the first bodybuilding magazine.^28^ Sandow placed less “emphasis on a healthful diet” than his great rival Bennett and shunned the use of any special foodstuffs. Despite this, in his 1897 exercise guidebook, *Strength and How to Obtain it*, he included a table listing the “Nutritive Qualities of Foods” and stressed the importance of letting at least two hours elapse between eating and exercising; this despite asserting in the same publication that he had “no belief in special diet.”^29^ For someone who claimed that following a particular diet was largely irrelevant to the purpose of maintaining a youthful, vigorous body, Sandow acknowledged surprisingly specific practices of eating, including Fletcherism.^30^ For example, he would

abjure anything intoxicating, confining myself mostly to beer and light wines. Tea and coffee I never suffer myself to touch. All I impose upon my appetites is that they shall be temperately indulged. I endeavour to have my meals at regular hours, and prefer that they shall be simple and easy of digestion. I always take care to chew my food, proper mastication being a sine qua non of health.^31^

Completing the Anglo-American trio of physical culture gurus who also attempted to assert their authority over what constituted a healthy diet designed to prolong youthfulness and vitality in the pre-vitamin era was Bernarr Macfadden. Macfadden, who established a publishing empire to promote his ideas, drew on his personal experience of the links between a sedentary lifestyle and poor health to establish the magazine *Physical Culture* in 1899. Like Bennett, Macfadden extolled the virtues of a vegetarian diet and periodic fasting as a means of achieving rejuvenation.^32^ Through his early publications Macfadden argued that “[t]here is but one way of creating a normal appetite and that is by fasting” and noted that, although vegetarianism was preferable, “I am not one of those who holds that a high degree of health cannot also be acquired and retained with a mixed diet.”^33^ By the early 1920s, in the context of emerging excitement about the nutritional potential of vitamins, Macfadden had reconfigured his arguments to provide a new rationalization for his system. For example, whereas in his 1901 book *Strength from Eating* he had concentrated on factors such as the “acid properties” of fruits which “are valuable in assisting the digestive process” and, like Sandow, “thorough mastication … until it [food] is an actual liquid,” later works drew on the language associated with vitamines.^34^ Macfadden presented his updated arguments in *Eating for Health and Strength*, first published in 1921. In this text, he adopted scientific terminology to support his system, claiming that “a single vitamine, which in quantity may be less than one thousand[th] of the weight of the food, is absolutely essential to life.”^35^ Further, and more pertinently for Macfadden’s aims as an entrepreneur:

Those of us who have studied the food question from a more practical, human standpoint had observed much evidence of the superiority of natural foods. The discovery of vitamines backs, with scientific fact and theory, these more practical human observations.^36^

Just as Macfadden’s work was reconfigured through multiple editions, other publications, such as Otto Abramowski’s simple and accessible pamphlet *Fruitarian Diet and Physical Rejuvenation* (1911) continued to be reprinted after the Second World War. In the rapidly-changing landscape of nutrition practices in the first half of the twentieth century, this manual for preserving health and youthfulness enjoyed remarkable longevity, even if its disciples did not experience similar effects. The text was published originally by the Order of the Golden Age. The Order was established in the mid-1890s by Sidney Hartnoll Beard and, through their various books and their periodical, *The Herald of the Golden Age*, sought to promote the cause of vegetarianism from a Christian perspective. Beard himself argued strongly that this diet should be pursued as a matter of urgent moral duty.^37^ When the Order relocated to South Africa in the late 1930s the organization quickly faded into obscurity, yet Abramowski’s work continued to appear in print. The success of his pamphlet – still recommended in some quarters of the vegetarian movement – was something of an exception to the relatively short-lived fashions of the period, when dietary innovations competed for public attention and scientific credibility.

Whilst Abramowski’s book dealt only tangentially with the subject of old age, F. W. D. Mitchell’s *A Key to Health and Long Life* (1914, second edition 1922) argued explicitly that “the most significant symptom of the approach of old age is the decline of the digestive powers.”^38^ Mitchell had begun his medical training in Dublin in the 1880s but withdrew despite successfully completing examinations in anatomy and physiology and by the early twentieth century occupied the post of Secretary of the Congested Districts Board in Ireland.^39^ Having sketched out a pre-vitamin system of dietary management in his initial publication in 1914, Mitchell then drew on the public attention afforded to the significance of accessory food factors in a subsequent edition eight years later. In this, he reserved special praise for “‘Vitamine’, which escapes chemical analysis and seems to be almost synonymous with the quality known as ‘freshness’.” Having asserted that fresh foods contained an abundance of this substance, Mitchell advocated a diet which shunned “salted or preserved foods” which could cause “the blood [to become] weak and unhealthy.”^40^ He echoed Bennett’s call to ensure that foods were cooked appropriately, as overcooking might destroy the essential “vitamin quality,” favoring fresh fruit and salads over cooked food, and castigating in particular margarine which he considered “deficient in the vitamines.”^41^

As Mitchell was not a medical professional he sought to bolster his credibility in a number of other ways. He drew heavily and explicitly in his own work on the writings of key authorities, including Cornaro, the celebrated English physician George Cheyne, Hermann Weber, Caleb Saleeby, Elie Metchnikoff, and also appealed to his own relationship with medicine by reprinting letters which he sent to the *Hospital Gazette* and *Medical Times*.^42^ His intervention – aimed at a non-professional audience – did not go unnoticed by the medical press. A review of the 1922 edition in the *British Medical Journal*, for example, remained critical of Mitchell’s arguments, yet also noted that “if the public for whom it is more suited read and follow its directions the result will be all to the good.”^43^

Many of the materials cited by Mitchell were produced by high-profile members of the medical profession. Hermann Weber (1823–1918), for example, argued specifically that vitamine was “destroyed by boiling,” particularly in the case of milk, which was otherwise considered to be “the most useful article of food … the most perfect food.”^44^ Weber had trained in Marburg and Bonn before settling in England to practice in 1854. He advocated environmental cures and health retreats in cases of consumption and turned his attention late in life to the relationship between diet and longevity, delivering a lecture on the subject to the Royal College of Physicians in 1903 at the age of eighty.^45^ This formed the basis of his book *On Longevity and Means for the Prolongation of Life* which ran through several editions before his death. Just as Bernarr Macfadden used emerging ideas about the role of vitamins in his later publications to provide new scientific justification for his earlier ideas, so too did later editions of Weber’s book retrospectively integrate such information into his existing arguments. However, far from identifying vitamins as all-powerful, they were but a small, yet important, part of Weber’s scheme of nutrition, which stressed above all the centrality of balance across the diet.^46^ In this regard, he drew analogies with the animal kingdom in order to demonstrate one of the key tenets of his argument which went against many of the self-proclaimed dietary experts of this period: that abstaining from flesh in the diet was not self-evidently positive for overall health. The rationale behind this was, as the prominent English eugenicist Caleb Saleeby, an adviser to the Minister of Food during the First World War, noted in his 1921 work *The Eugenic Prospect*, that “the herbivore, eating the green leaf, does well; and the carnivore, eating the herbivore, does no worse.”^47^ This, Saleeby and Weber maintained, was because the leaves were the true site of vitamin generation, and these remarkable substances simply made their way up the food chain in an uncomplicated fashion.

In earlier editions of his work, Weber placed great emphasis on the purity of milk as a foodstuff. For the purpose he drew largely on the assertion of one of the most significant figures in the longevity movement of the early twentieth century, Elie Metchnikoff (1845–1916). Writing before the First World War, Metchnikoff almost singlehandedly initiated the idea that “beneficial microorganisms should be introduced into the colon to fight the toxicity” produced by the digestive process.^48^ This toxicity, he argued, which arose from decomposition, was the cause not only of intestinal complaints, but also the aging process.^49^ To combat these destructive influences, Metchnikoff advocated the consumption of yogurt, the natural bacterial content of which would modify the internal environment in such a way as to prevent the build-up of toxins to a dangerous level.^50^ However, although the regular consumption of yogurt was a unique feature of his approach to maintaining a healthy diet – and consequently remaining youthful – Metchnikoff also drew on similar practices of abstention and dietary restraint as those promoted by the likes of Weber and Saleeby. These included “[t]he avoidance of alcohol and the rigid exclusion from diet of foods that favour putrefaction, such as rich meats.” The ultimate goal of Metchnikoff’s system of dietary modification was nothing less than extension of the average lifespan, and he cited the prominent role played by soured milk foods in a number of national contexts, from the “Leben raib” (a soured milk prepared from the milk of buffaloes or goats) of Egypt to “prostokwacha” (coagulated, soured, raw milk) consumed in Russia.^51^ As he summarized: “[t]he fact that so many races make soured milk and use it copiously is an excellent testimony to its usefulness.”^52^

Metchnikoff implicated germs of disease, albeit indirectly, in the aging process. However he did so in a way which reinforced traditional narratives about the desirability of a simple diet, especially the avoidance of heavy foods and adherence to the “rules of rational hygiene.”^53^ These ideas were not displaced, but rather reinterpreted in the era of vitamins which followed the First World War; a simple, raw diet of fresh, natural foods was now beneficial for preserving health and rejuvenating body and mind by virtue of its vitamin content. Metchnikoff’s work is frequently placed alongside the endocrine researches of two other advocates of rejuvenation and regeneration in this period: Eugen Steinach and Serge Voronoff. Both emphasized the possibilities afforded by surgical treatments – vasectomy in the case of Steinach, for Voronoff testicular grafting – for combating the onset of senescence. Their significance in the history of hormones, rejuvenation and wider interwar society has been much-discussed, but it is telling that Voronoff in particular included limited references to diet in his writings.^54^

**Figure 1.**
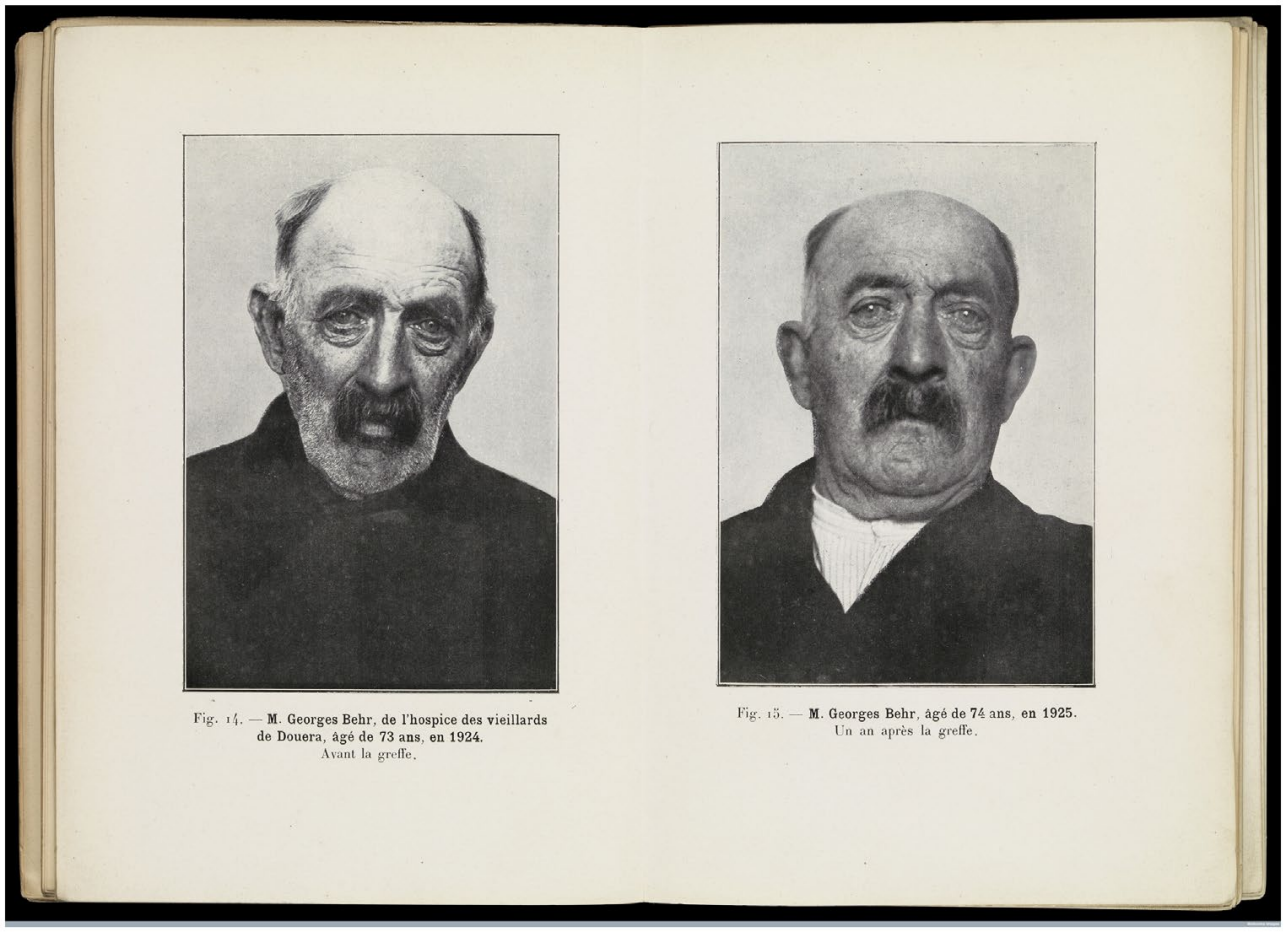
A characteristic image of claimed rejuvenation, published in one of Serge Voronoff’s most complete accounts of his methods. Notes: The patient, Georges Behr, is pictured prior to treatment at the age of 73 and one year later after the gland-grafting operation which gained much notoriety throughout the Western world in the interwar period. Serge Voronoff, *Étude sur la vieillesse et la rajeunissement par la greffe*, Paris: G. Doin, 1926, reproduced by kind permission of Wellcome Library, London, L0068506.

Alongside the return of sexual potency, chief among the claims made by both Steinach and Voronoff was the relationship between rejuvenation by hormonal means and the return of increased appetite.^55^ As one of Steinach’s own patients reportedly said, shortly after the procedure “[my] appetite began to increase to such a degree that I could hardly satisfy it.”^56^ The connections between successful rejuvenation by hormone treatments and increased appetite became a key feature of the public discourse, and the before-and-after images used by Voronoff in his publications provided clear visual evidence for readers that his rejuvenated subjects had gained rather than lost weight following the treatment (see Figure 1). Voronoff himself did not implicate vitamins in the ageing process, although he did note that dietary habits more generally had a (limited) effect on longevity.^57^ As well as focusing on weight gain as a marker of successful rejuvenation, Voronoff also mobilized biological authorities such as Georges-Louis Leclerc, Comte de Buffon, to argue that “the duration of life does not depend on habit, custom or quality of food.”^58^

Voronoff denied that there was any particular link between diet, lifestyle and longevity on the grounds that

though we discover amongst those who have passed the century mark abstemious persons who content themselves with bread, milk, and a vegetable diet, and avoid excess in all things, we find among them many individuals who have led a wild life, who have been very heavy drinkers, or have misused tobacco, coffee, and other stimulants and drugs.^59^

As if to cement the point, when Voronoff’s British supporter, Leonard Williams, delivered his Cavendish Lecture to the West London Medico-Chirurgical Society on 27 May 1927, he articulated an important role for vitamins in normal physiology and maintenance of health, but one which was quite separate from the action of the endocrine system.^60^

## Authority and Testimony: Promoting Diet

It is hardly surprising that Voronoff had little to say about diet and did not mention vitamins at all. After all, a central aspect of his promotion strategy was to emphasize the all-pervasive power of hormones. However, as we explore in this section, would-be dietary authorities sought to mobilize modern understandings of the body and vitamins alongside established promotional devices such as personal testimony. Whilst some articulated a vision of diet and the endocrine system working in tandem, others maintained the primacy of diet over the surgical interventions suggested by Voronoff, Steinach and their advocates.

Caleb Saleeby, who we have already encountered in his role as an advocate of an omnivorous diet, was also a founding member of the New Health Society in 1925 alongside William Arbuthnot Lane, whose role in this organization has been well-documented.^61^ Lane and his associates used the Society’s journal, *New Health*, as a means of disseminating an approach to health and social medicine rooted in digestion, shunning surgical intervention. This marked a considerable departure for Lane, who had earlier argued in favor of removing large sections of the digestive tract as a cure for constipation.^62^ For Lane and numerous contributors to *New Health*, the discovery of vitamins and their role in the maintenance of normal, healthy bodily function, represented an important opportunity to claim authority to speak on matters of everyday health and wellbeing. One such author, the noted American naturopath Benjamin Gayelord Hauser, castigated the methods of Voronoff and Steinach for producing, at best, temporary results. Instead, he urged readers to cultivate “healthy glands through a vitamin-rich diet.”^63^ Hauser, who had been treated early in his life by the prominent American naturopath Benjamin Lust, opposed excessive consumption of white bread and sugar in common with many of his contemporaries. This was based on the high vitamin content of the wheat germ – absent in white flour – and the vacuous nutritional nature of refined sugar. Further, he also posited particular roles for specific vitamins, which would “aid the health of the glands”: Vitamin D maintained thyroid gland function, Vitamin B regulated the pituitary gland, and Vitamin E acted on the sex glands.^64^ In both his articles for *New Health* and many popular books on natural eating, Hauser therefore argued that the key to maintaining the condition of the glands in older age – and therefore prolong youthfulness – lay in diet, not surgery. For instance, his 1930 guide designed to “mould our bodies or restore youth,” *Harmonized Food Selection*, exhorted the reader to indulge in “radiant, life-giving foods” such as olive oil and lemon juice which would “feed the glands” and remove impurities.^65^

The links between glandular health and diet, explored explicitly by the likes of Hauser, was also reinforced by some of the most prominent public discourse surrounding rejuvenation. The connections between ageing and eating reinforced in a new and arresting way the importance of correct diet for youthfulness and longevity. At the confluence of the relationship between glands and vitamins were organizations such as the British-based Pharmaceutical Research Institute – in reality a quasi-medical manufacturer of gland extracts – which advertised through newspapers a seemingly unique form of therapy, which combined “[g]landular therapy, without injection, in conjunction with a vitamin catalyst,” hailed as a triumph of modern “medical science.”^66^

Although many sought to emphasize the link between glandular activity, appetite, and rejuvenation, others dismissed such connections in favor of monolithic explanations of the aging process. Personal testimony extolling the virtues of withholding food from the body promoted periodic fasting as a sure-fire way of maintaining youthfulness and vitality for longer. Many of these claims were a direct reaction against high-profile surgical rejuvenation methods. For example, Mr. Tuohy, described by the *Bedfordshire Times* in 1921 as a “veteran faster,” was “no believer in the grafting of glands as a means of rejuvenation … Fasting is much more economical for the simple reason that to go without food costs nothing and gives the body a rest.”^67^

Similar accounts of youth restored through a combination of fasting and dietary modification abounded. Mr. Bate, for example, who was “nearly 70,” took radical action when his doctor told him that “old age was coming on fast.”^68^ After a fast of some forty-six days, Bate then “lived entirely on raw fruit and vegetables,” echoing almost exactly Hermann Weber by arguing that “when you boil a cabbage you destroy its body-building properties.”^69^ Bate did not adopt the language of vitamins; similar accounts using so-called “plain” speak came from others who sought to use their personal experience to plot links between diet and ageing. For example, in the late 1920s a Mrs Rogers from Eastbourne in southern England gave a number of vegetarian cookery demonstrations, arguing that “vegetarians claimed that by their dietary they could rejuvenate themselves.”^70^ The specific language associated with vitamins was therefore an important but not essential part of dietary discussions in the public domain through the 1920s. The more nebulous terminology of various so-called “properties” – vital, building, and so on – persisted in public discourse around how diet might maintain bodily youthfulness.

As well as the personal testimonies of successful exponents of rejuvenatory diets, in Britain public health officials also dispensed advice about how to maintain health and maximize healthy lifespan. In 1925, for example, the Medical Officer of Health for Deptford, Charles S. Thompson, urged visitors to the major international Food Exhibition at Olympia to modify their eating habits by “giving up the English breakfast for the French breakfast of coffee and a roll.”^71^ According to Thompson, “[a]fter forty there must be no more beer,” and “a man of forty-five or fifty – and, dare one add, a woman? – should not indulge in the foolish afternoon tea with cake, bread, and butter.”^72^ Taken together, the advice was aimed at tackling the increasing anxiety about Britain becoming a “sparsely populated country where the young-old men dwell.”^73^

The dietary wisdom of a majority of public health officials and almost all self-proclaimed nutrition experts in their writings – occasionally promoting moderation, more frequently a shift to a diet characterized by a high proportion of fruit and vegetables, and often radical reduction in consumption through fasting – represented a form of “soft” everyday rejuvenation at odds with the “hard” surgical intervention which brought notoriety to the glands as the primary governors of youthfulness. However, all sought to promote healthy habits of eating as either an important cause or effect of rejuvenation. For some – such as Macfadden, Bennett, and Hauser – commercial imperatives underpinned all their activities. For others, particularly in Britain, the aftereffects of rationing during the First World War placed a practical, economic necessity on modifying diet at a national level; interventions of this kind – spearheaded by government and public health officials are, however, beyond the scope of this paper. We move now to consider how manufacturers of vitamin-containing food supplements drew on the language and popularity of rejuvenation to exploit a new marketplace for enriched foods and extracts which claimed to prolong youthfulness and vitality.

## A New Age of Vitamins and Consumerism

By the 1930s the vitamin content of food, alongside its mineral and caloric composition and long-established categories of protein, fat and carbohydrate, was widely acknowledged to be a key factor in determining its nutritional value. The implications for diet were obvious: the specific functions of different vitamins and the health requirements of the individual dictated the foods which they should eat. Following a decade in which rejuvenation of both individual and nation had become an object of societal fascination, these still-imperfectly-understood substances were endowed with extraordinary capacities to modify human physiology and intervene in the ageing process. However, vitamins were taken up in a still wider range of ways and were presented by various commercial groups as integral components of lifestyle and health beyond diet. As Rima D. Apple has argued, in the United States the early debate was not “who could sell vitamins,” but “who could judge vitamin supplementation.”^74^ This was manifestly not the case in early twentieth-century Europe, where the food supply to tables was affected significantly as the proximity of conflict had a far more profound nutritional effect. In this context, vitamins functioned as an “advertiser’s dream”; however, this was just as much linked with aging as nutrition.^75^

Manufacturers attempted to capitalize on the appeal of vitamins to sell specific products, such as Fleischmann’s Yeast, Quaker Oats, and Welch’s Grape Juice, many of which emphasized their place in a diet which promoted vigor and preserved health.^76^ In the case of Fleischmann’s Yeast, promotional material even claimed that it was naturally “rich in hormone-like substances.”^77^ However, others sought to integrate vitamins within a broader vision of rejuvenation. The Théiron School of Life, an organization based (in Britain at least) initially in Bond Street, London, later in Henrietta Street and the less salubrious St. Martin’s Lane, and which offered a correspondence course in “how to remain young and achieve, how to develop a pleasing personality [and] how to overcome ‘old-age’” was one of a range of organizations which promoted rational healthy eating as part of a system which enabled adherents to reach these lofty goals.^78^ Although scant records of the Théiron School remain, newspaper advertisements and published courses aside, the practical guides, promotional materials and study questions for subscribers offer a fascinating insight into a little-studied organization which promised to “give you back youth, beauty and vitality” with “[n]o operation, no danger.”^79^ It seems that the enterprise originated in 1930 with the scientific endeavor and personal experience of a “professeur Théiron” in Brussels, who drew on the transformative and controversial cellular immortality studies of Alexis Carrell to claim that, given the correct environmental conditions, life could be postponed dramatically beyond its current limits.^80^

Diet was central to the approach of the Théiron Method and strongly connected with both the glandular system and aging.^81^ As one of the introductory sections on “Scientific Feeding” explained:

… when one considers that the glandular system determines one’s personality, and that these glands require certain chemicals which are frequently absent from the diet, or eaten in a haphazard sort of way, there is no doubt that the character is more or less determined by what one eats.^82^

Much of this hinged on chemical elements as constituent parts of food, underpinned by claims to scientific authority:

The new science of bio-chemistry demonstrates that a man who takes insufficient oxygen into his system, develops a depressed and hopeless outlook in life … A man who eats food containing an excess of carbon becomes incurably lazy, mentally stupid, and loses ambition. … Those people who do not secure sufficient hydrogen take on the appearance of premature age.

An impact and influence on personality or health was identified for almost every conceivable element, including nitrogen (“magnetic” personality), phosphorous (intelligence), sulphur (excitability), sodium (restlessness), “chlorin” (gloom), “silicum” (baldness) and magnesium (sensitivity to heat). Whilst the student of the Théiron Method was introduced to these more traditional, “mineral” components of food, special place was reserved for “certain chemical combinations, which we have termed “vitamins,” [which have] opened up an entirely new field of dietetics.”^83^ The fact that these were, in the case of cereals, for example, located principally in “the hull or outer covering” led the method to classify refined white bread as inferior, just as Hermann Weber, Benjamin Hauser and others had done a decade earlier on almost exactly the same grounds.^84^

The Théiron Method was unequivocal in rejecting the consumption of “flesh food,” which was considered to be “not for humans.”^85^ However, the diet suggested was claimed to be distinctive from vegetarianism, which led to “lack of health and vitality … sallow skin and other unwholesome symptoms.”^86^ The rationale for this was that the Théiron School advocated eating only sun-ripened vegetables, as these contained the greatest proportion of vitamins A, B, and D. Turnips, beetroots, and other root vegetables were considered off-limits – adherents were advised to “feed these vegetables to your cow” – while the dietary advice was also tailored according to complexion.^87^ Happily for those of a fair disposition, in the case of ice cream, “blondes can eat it and enjoy it without much harm to themselves,” while “[b]runettes who feed upon ice-cream, and drink iced drinks … interfere with their digestion, and bring about an unwholesome condition sooner or later.”^88^ Even many of the external signs associated with aging were attributed to dietary indiscretion: “[o]ne of the most important causes of greying hair is wrong diet.”^89^

As a means of promoting health, the modified vegetarian diet advocated by the Théiron School was also rooted in claims of a scientific nature. For example, the relatively short alimentary canal of humans compared to flesh-eating animals, the fact that most “carnivorous animals are active at night” and the lack of teeth or claws “with which to tear flesh” all provided justification for taking such an approach to diet.^90^ In the competitive interwar landscape of both complementary and conflicting methods of rejuvenation, the ability to speak authoritatively on matters of human physiology and pathology was essential for success. Advocates of almost all products and procedures made some appeal to expertise in this regard, and both new and existing approaches now claimed to be rooted in emerging forms of scientific knowledge.

It is difficult to say with any certainty exactly what impact the Théiron Method had on the everyday practices of rejuvenation through diet and exercise. However, two things are certain. First, as discussed, in the expensively produced and lavishly illustrated publications emanating from the School, vitamins played a key role in anchoring theories of rejuvenation and youthfulness. Second, the School itself came to the attention of one of the most prominent commentators on rejuvenation from the period, Maurice Ernst, with promotional materials appearing in his papers.^91^

At the same time as organizations like the Théiron School were promoting the youth-making properties of certain approaches to diet, the apparent ability to produce refined (or, it was even claimed by some, synthesized) vitamins – known invariably as “extracts” – saw the reconfiguring of the commercial landscape of dietary supplements. Products which had previously claimed to treat more generalized nervous disorders, characteristic of the interwar period, were now marketed by manufacturers as guardians of youthfulness, vitality and energy. Many of these drew on the supposed universal power of vitamins, linking deficiency to the premature onset of old age. One such product was Vitamina, the “secret of new youth at any age,” which was developed by the “French savant” and disciple of Professor Berthlot, Dr Paulin.^92^

Marketing materials emphasized Paulin’s scientific credentials alongside the designation of Vitamina as “not a drug but [a] natural food of the highest order,” which:

introduces Vitamins and Mineral Salts into the Blood Stream in the correct proportions found in Nature … rendering the blood beautifully clean and fluid. This improved Fluidity at once enables the Heart to circulate it, without effort, into the furthermost tissues and automatically build Strong Nerves and Radiant Health.^93^

In Britain, two products of the late interwar period which also emphasized their natural origins were Vikelp and Oystrax. The former – “a concentrated extract of a Pacific Ocean plant” – was claimed by its manufacturers to be “the richest known source of 9 of the 12 minerals essential to health and well-being” as well as containing critical “vitamins and food iodine” considered equally valuable.^94^

Oystrax, a similar product which contained “raw oyster stimulants, vitamins [and] general invigorators,” especially “Vitamin E, the ‘gland-toning’ Vitamin,” claimed that both men and women past the age of forty could “feel as young as ever.”^95^ First advertised in 1937 through local newspapers in Britain, they continued to be marketed as restorers of vigor until the early 1950s, reflecting a continuing general anxiety around the Second World War about the energy and fitness of the British populace.^96^ These products promised a restoration of youthfulness and vitality, making accessible to a wider audience the rejuvenatory possibilities of “glandular re-fuelling treatments.”^97^

One of the most enduring products of this kind was Phyllosan. Described in some of the earliest newspaper coverage as “a new extract in tablet form of watercress, spinach and other green plants” which might be a “rival to lime juice in preventing scurvy,” it persisted in the marketplace for almost fifty years.^98^ Like many products which became successful in the public sphere, Phyllosan started life in the clinical environment; in this case that was at “several London hospitals, where patients have been rejuvenated by a course of treatment.”^99^

In common with many personal accounts of rejuvenation, Phyllosan was lauded for its ability to produce “rejuvenation without operation.” The promotional literature contained a curious mix of humoral theories of bodily regeneration which co-existed with chemical symbols explaining how Phyllosan was able to exert such a remarkable effect on the body:

Phyllosan is certainly the greatest specific yet discovered for enriching the blood … and eliminating the *physical signs of advancing years*. By its remarkable chemical structure, Phyllosan possess the properties of restoring to normal all physical and vital forces … The composition is represented scientifically in the formula (C16, H18, N2O).^100^

Among the beneficial effects were an improvement “in general health … an increase in appetite, energy and the joy of life.”^101^ Aimed squarely at those over the age of forty – an advertising campaign that ran for well over a decade claimed that it would “fortify the over-forties” – Phyllosan was arguably the most successful everyday rejuvenation product of the period.^102^ Its ubiquity and public visibility, continuing in the post-war period, forces us to challenge the dominant historical narrative that public discourse surrounding rejuvenation was largely based around hormones and the endocrine system.

## Conclusion: Fasting, Rational Diet, and the Negotiation of Expertise

[N]atural uncooked food has saved my life, has rejuvenated my body and made out of an over-fed, old man, courting apoplexy and rushing blindly into a premature grave, a comparatively young, vigorous and healthy person, fit and willing to live another half century.^103^

By the early twentieth century, middle age was seen as a time of physiological crisis rather than intellectual flourishing.^104^ In response to this the restorative, vegetarian fasting programs advocated by Bennett and Macfadden, and the moderation promoted by Sandow took on a new sense of urgency: repairing and purifying the body was a moral, social duty. This was heightened still further in the aftermath of the First World War when national fitness and economic productivity became focal points of public and professional debate. We know that conflicts of the twentieth century had a profound impact on diets, both in the European regions and more widely.^105^ In this context, the ability of the digestive system to extract nutrition from food easily and efficiently became inextricably linked with age and youthfulness. Even in the United States, where formal rationing was relatively limited, Helen Zoe Veit has argued that food promotion, propaganda and consumption was one of the most prominent articulations of Progressivism around the period of the First World War.^106^ As I have explored here, the interwar period saw an unprecedented land-grab for dietary authority. This was precipitated on the one hand by the discovery of accessory food factors – vitamins – and on the other by far-reaching social change which emphasized the importance of maintaining healthy, fertile, and economically productive citizens. Although public discussion surrounding the latter was cast in terms of the centrality of hormones, exercise, and other factors alongside diet, vitamins were also strongly bound up with mechanisms of bodily control and the aging process, lending new support to both fringe and mainstream theories about the relationship between diet and longevity.

Medical professionals, self-styled dieticians, entrepreneurs, dietary adherents themselves, and a range of other actors promoted a host of approaches to eating, many of which attempted to establish scientific credibility to substantiate their claims to bring about rejuvenating effects on the individual. These ranged from the moderate pluralism of an omnivorous diet to period fasting, and from endless variations of vegetarianism to the consumption of sour yogurt. Almost all relied to some extent on the supposed power of vitamins in human physiology, and many also sought to capitalize on the apparently sensational higher bodily power of the ductless glands and hormones.

Returning to the question with which we began, in an era of widespread anxiety about the health, fertility, and fitness of both individuals and nations, what role did innovations in dietary science and practice – especially vitamins – have in everyday attempts to combat aging? On the one hand the discovery of the importance of vitamin content in various foodstuffs simply provided another means of justifying long-established dietary practices, many of which were linked to moral positions. Prominent among these were the need to combine a diet of moderation with exercise, especially that rooted in physical culture, and the superior nutritional qualities of raw or very lightly cooked food. However, the strong connection between appetite and the glandular system also saw the emerging specialism of endocrinology raise the prospect of a reinterpretation of desirable eating habits, especially the maintenance of a larger appetite at an older age and the rejection of specific foods such as white bread.

The mainstream medical profession was largely united in its adherence to a somewhat conventional, pluralistic diet, yet at the same time a significant coalition of individuals and organizations, who emerged from causes ranging from anti-vivisectionism and physical culture to eugenics and religion, championed the mission of vegetarianism, itself a diverse set of dietary practices. As well as various ideological motivations, these debates were further crystalized by the development of commercial interests which recognized the potential to market vitamin extracts and vitamin-rich supplements as essential components of a healthy diet. In among this, self-proclaimed dietary authorities competed against one another. Proponents of “hard” rejuvenating treatments and devices such as Voronoff’s gland-grafting and Steinach’s vasectomy noted with satisfaction the restoration of hearty appetites as an indication of success, whilst others who claimed that rejuvenation could be achieved in a “softer” fashion – often through a combination of dietary modification and exercise – maintained the importance of moderation and abstention.

At the heart of these different perspectives was a battle not just for scientific authority, but for moral territory associated with the promotion of both a healthy lifestyle and a temperate demeanor during a turbulent period across Western society. The constitution of an optimum diet was, for some, transformed by the discovery of vitamins and their effects on the body. For others, an awareness of the vitamin content of foods simply provided a renewed justification for retaining existing dietary habits. Vitamins themselves therefore had a lively existence both within and beyond the dining room as they vied with hormones, electricity and other non-naturals to be seen as the most essential components of a youthful, healthy body.

## Funding

This work was supported by an AHRC Leadership Fellow award [Ref. AH/N007735/1]; a Wellcome Trust Small Grant [Number 104729/Z/14/Z].

## Notes on Contributor

***James F. Stark*** is Associate Professor of Medical Humanities at the University of Leeds. He is primarily a historian of modern science and medicine, and his other work includes a book-length trans-national history of anthrax: *The Making of Modern Anthrax* (London: Pickering and Chatto, 2013). He has published in *Medical History*, *Social History of Medicine*, *Museum and Society*, and *Heritage and Society*, and edited special issues of the *British Journal for the History of Science* (“Owning Health”, 2016) and *Palgrave Communications* (“Human Regeneration”, 2018).

## Notes

1. Medical Research Committee, *Report on Accessory Food Factors*. A short summary of the report’s major findings was also published in *The Lancet*: “Accessory Factors in Food.”

2. Hopkins, “The Analyst.”.

3. Hopkins, “Feeding Experiments.”

4. In his 1921 Chandler Medal Address, given at the University of Columbia, Hopkins sought to downplay his own role in early identification of accessory food factors, noting that he had “received credit – perhaps too much credit – as pioneer in their discovery.” Hopkins, *Newer Aspects*, 14. Even in the early 1920s Hopkins argued that the term “vitamine” was misleading since the precise nature of the substance(s) – thought by Funk to be amines – remained uncertain. See Eddy, *The Vitamine Manual*, 10.

5. See, e.g. Boeke, “The Histological Basis,” 221.

6. Gowland Hopkins was awarded the 1929 Nobel Prize with Christiaan Eijkman for their collective work on vitamins, although Hopkins’ contribution was cited as the “discovery of the growth-stimulating vitamins.” “Nobel Prize 1929”. See also Kamminga and Weatherall, “Making of a Biochemist I,” 275; Hopkins, *Newer Aspects*, especially 17–18; Hopkins, “Discussion on vitamin-A.”

7. Medical Research Committee, *Report on Accessory Food Factors*, 1. See also Drummond, “Aspects of Infant Feeding”; Dale, “Edward Mellanby”; Hopkins and Martin, “Arthur Harden.”

8. Kamminga and Weatherall, “Making of a Biochemist I,” 275.

9. Sengoopta, “Dr. Steinach.”

10. Miller, *History of the Stomach*. Miller argues that concerns over diet across the early twentieth stemmed from nationally bound anxieties such as increasing urbanization and represented an extension of late nineteenth century preoccupation with the problem of degeneration.

11. Horrocks, “The Business of Vitamins”; Horrocks, “Nutrition Science”; Oddy, *From Plain Fare*; Vernon, *Hunger*; Biltekoff, *Eating Right*.

12. Buckley, “Recipe for Reform”; Shprintzen, *Vegetarian Crusade*; Kamminga and Cunningham, *Science and Culture*; Kamminga, “Axes to Grind”; Carpenter, *Beriberi, White Rice*; Carpenter, *Scurvy and Vitamin C*; Biltekoff, *Eating Right in America*; Gilheany, *Familiar Strangers*.

13. McGrady, *Youth Doctors*; Trimmer, *Rejuvenation*.

14. See, e.g. Boia, *Forever Young*; Botelho and Thane, *Women and Ageing*; Haycock, *Mortal Coil*; Hirschbein, “The Glandular Solution”; Hoberman, *Testosterone Dreams*; Logan, *Hormones, Heredity and Race*; Johnson and Thane, *Old Age from Antiquity*; Ottaway, *Decline of Life*.

15. Lloyd George, *Looking Forward*, 3; London County Council, *Annual Report*, 4.

16. For practical reasons, I reserve any substantive discussion of gender for other publications.

17. Zweiniger-Bargielowska, *Managing the Body*.

18. For a recent example of transnational dietary innovation, see: Haushofer, “Between Food and Medicine.”

19. Bennett, *Old Age*, 28.

20. Ibid., 30, 31; Bennett, *Exercising in Bed*.

21. Lorand, *Health through Rational Diet*, 398. Lorand’s other works included a more general treatise on combating old age, *Old Age Deferred*, as well as *Life Shortening Habits and Rejuvenation*.

22. Bennett, *Old Age*, 135.

23. Ibid., 189.

24. Ibid., 18, 19.

25. Martschukat, “The Necessity for Better Bodies”; McKenzie, *Getting Physical*.

26. Bennett, *Old Age*, 26, 126.

27. Zweiniger-Bargielowska, “The Culture of the Abdomen,” 242.

28. Scott, “Body-building and Empire-building.”

29. Chapman, *Sandow the Magnificent*, 175; Sandow, *Strength and How to Obtain it*, 13, 35, 90.

30. Derived from Horace Fletcher, Fletcherism was a system of eating based on chewing food until it had become liquid and thereby easier to digest. See Christen and Christen, “Horace Fletcher.”

31. Adam, *Sandow on Physical Training*, 164, 165.

32. Griffith, “Apostles of Abstinence.” See also Ernst, *Weakness is a Crime*; Adams, *Mr. America*; Endres, “The Feminism of Bernarr Macfadden.”

33. Macfadden, *Strength from Eating*, 73.

34. Ibid., 177, 188.

35. Macfadden, *Eating for Health and Strength*, 21.

36. Ibid., 45, 46.

37. Beard, *Flesh*-*eating*; Beard, *Comprehensive Guide*-*book*; Bates, *Anti*-*Vivisection*, 69–98.

38. Mitchell, *Key to Health*, 163.

39. “Medical News,” 973; Royal Commission on Congestion in Ireland, *Minutes of Evidence*, 22; Breathnach, *The Congested Districts Board of Ireland*.

40. Mitchell, *Key to Health* 2nd edition, 115.

41. Ibid., 115, 116. Macfadden made a similar argument, noting that “[t]he cooking process may destroy or reduce the available quantity of vitamines [in leafy green vegetables].” Macfadden, *Eating for Health and Strength*, 152.

42. These letters were included as appendices in both the 1914 and 1922 editions. Mitchell, *Key to Health* 1st edition, 151–9.

43. “Notes on Books,” 1179.

44. Weber, *On Longevity*, 87, 89.

45. Weber and Weber, *Climatotherapy and Balneotherapy*.

46. Weber, *On Longevity*, 80, 81.

47. Saleeby, *Eugenic Prospect*, 183.

48. Stoilova, “Bulgarianization of Yoghurt,” 19.

49. Later proponents of this view included William Arbuthnot Lane. See Lane, “Chronic Intestinal Stasis.”

50. Metchnikoff, *Prolongation of Life*. Some authors have argued that this approach was the defining precursor to modern practices associated with probiotics, notwithstanding earlier pre-germ approaches to cultivating gut flora. See, e.g.: Mackowiak, “Recycling Metchnikoff”; McFarland, “Yaks to Yoghurt”; Tauber and Chernyak, *Metchnikoff.* John Harvey Kellogg was another high-profile advocate of the important role played by auto-intoxication from diet. See Kellogg, *Autointoxication*.

51. Metchnikoff, *Prolongation of Life*, 171, 172.

52. Ibid., 174.

53. Ibid., 183.

54. Sengoopta, *Most Secret Quintessence*.

55. Krementsov, *Revolutionary Experiments*, 131; Steinach, *Verjüngung*; Sengoopta, “Rejuvenation and the Prolongation,” 59–61.

56. “Secret of Youth,” 3.

57. Nanninga, *Gland Illusion*, 67.

58. Voronoff, *Life*, 5.

59. Ibid., 8, 9. Nanninga takes the first part of this quotation out of context, and quite wrongly claims that Voronoff argued for an important link between diet and longevity. Rather, he was keen to highlight a relationship between *appetite* and vitality as a means of demonstrating successful rejuvenation. Nanninga, *Gland Illusion*, 67.

60. Williams, “Senescence and Senility,” 1179 and 1181.

61. Zweiniger-Bargielowska, “Raising a Nation.”

62. Lane, *Operative Treatment*; Lane, *The Results of the Operative Treatment*; Whorton, *Inner Hygiene*.

63. Hauser, “Prolong Youth,” 9. For a more discursive work by Hauser, see *Look Younger, Live Longer*.

64. Hauser, “Prolong Youth,” 10.

65. Hauser, *Harmonized Food Selection*, 31.

66. “A Million Years of Youth.”

67. “Interview with Mr. Tuohy.”

68. “Rejuvenated at Age of Seventy.”

69. Ibid., 1.

70. “For the Vegetarian.”

71. “The Search for Youth.”

72. Ibid., 6.

73. Ibid., 6.

74. Apple, *Vitamania*, 84.

75. Levenstein, *Revolution at the Table*, 152. Levenstein does not consider the characterization of vitamins in relation to the aging process, and other authors only mention this briefly in passing. See, for example: Scrinis, *Nutritionism*, 188.

76. Levenstein, *Revolution at the Table*, 153.

77. Ibid., 197.

78. Théiron School of Life, *Théiron Method*, title page; “Youth, Personality and Achievement”; “I Would Not Accept £100”; “Don’t Grow Old.”

79. “Personal.”

80. “Une Methode Intelligente de Vivre.”

81. Matthew Thomson, who provides a very brief reference to the Théiron School (with reference only to a later, more general textbook on practical psychology from 1936), suggests that this was an example of “practical psychology”, an approach which gained popularity particularly in the 1930s. However, that is to misrepresent the Théiron method which relied as much on nutrition and exercise as what Thomson terms the use of “subconscious mental powers”. See: Thomson, *Psychological Subjects*, 47 fn. 186.

82. Théiron School of Life, *Théiron Method*, 143.

83. Ibid., 251.

84. Ibid., 251; Weber, *On Longevity*, 98.

85. Théiron School of Life, *Théiron Method*, 196.

86. Ibid., 255.

87. Ibid., 255.

88. Ibid., 260.

89. Ibid., 529.

90. Ibid., 196.

91. Evans, “The Problem of Death,” 19.

92. Advertisement, *Daily Herald*, May 2, 1939. Berthelot was one of the most prominent chemists of the late nineteenth century, and by the 1890s he claimed that chemistry would eventually enable the synthesis of all foodstuffs, replacing natural products just as the new science of electricity would ultimately render gas obsolete.

93. Advertisement, *The People*.

94. Advertisement, *Daily Herald*, October 13, 1939.

95. Advertisement, *Liverpool Evening Express*; Advertisement, *Yorkshire Evening Post*; Advertisement, *Manchester Evening News*; Advertisement, *Sheffield Evening Telegraph*.

96. For the last advertisement in the British press, see Advertisement, *Aberdeen Evening Express*.

97. Advertisement, *The Daily Mirror.*

98. “Rival to Lime Juice.”

99. Ibid., 10.

100. “Rejuvenation Without Operation.”

101. Advertisement, *Daily Record*.

102. For early and late examples of the advertising campaign, see: “Are you Over Forty?”; “Phyllosan.” The “fortifies the over-forties” campaign was instigated in 1945, but Phyllosan was marketed at this demographic from its origins in the 1920s.

103. Abramowski, *Fruitarian Diet*, 1.

104. Heath, *Aging by the Book*.

105. Prodohl, “Dinner to Dynamite.”

106. Veit, *Modern Food*; Nordstrom, “And Serve the Cause.”
